# Expression profiles and function prediction of tRNA-derived fragments in glioma

**DOI:** 10.1186/s12885-023-11532-8

**Published:** 2023-10-20

**Authors:** Deng Wei, Ben Niu, Bei Zhai, Xiao-bai Liu, Yi-long Yao, Chan-chan Liang, Ping Wang

**Affiliations:** 1 Department of Neurobiology, School of Life Sciences, China Medical University, Shenyang, 110122 China; 2 Key Laboratory of Neuro-oncology in Liaoning Province, Shenyang, China; 3 Department of Neurosurgery, Shengjing Hospital of China Medical University, Shenyang, 110004 China

**Keywords:** Glioma, tRNA-derived fragment, tRF, Non-coding RNA, S100A11

## Abstract

**Background:**

Glioblastoma (GBM) is the most aggressive malignant primary brain tumor. The transfer RNA-derived fragments (tRFs) are a new group of small noncoding RNAs, which are dysregulated in many cancers. Until now, the expression and function of tRFs in glioma remain unknown.

**Methods:**

The expression profiles of tRF subtypes were analyzed using the Cancer Genome Atlas (TCGA)-low-grade gliomas (LGG)/GBM dataset. The target genes of tRFs were subjected to Gene Ontology, Kyoto Encyclopedia and Gene set enrichment analysis of Genes and Genomes pathway enrichment analysis. The protein-protein interaction enrichment analysis was performed by STRING. QRT-PCR was performed to detect the expressions of tRFs in human glioma cell lines U87, U373, U251, and human astrocyte cell line SVG p12. Western blot assay was used to detect to the expression of S100A11. The interaction between tRF-19-R118LOJX and S100A11 mRNA 3’UTR was detected by dual-luciferase reporter assay. The effects of tRF-19-R118LOJX, tRF-19-6SM83OJX and S100A11 on the glioma cell proliferation, migration and in vitro vasculogenic mimicry formation ability were examined by CCK-8 proliferation assay, EdU assay, HoloMonitor cell migration assay and tube formation assay, respectively.

**Results:**

tRF-19-R118LOJX and tRF-19-6SM83OJX are the most differentially expressed tRFs between LGG and GBM groups. The functional enrichment analysis showed that the target genes of tRF-19-R118LOJX and tRF-19-6SM83OJX are enriched in regulating blood vessel development. The upregulated target genes are linked to adverse survival outcomes in glioma patients. tRF-19-R118LOJX and tRF-19-6SM83OJX were identified to suppress glioma cell proliferation, migration, and in vitro vasculogenic mimicry formation. The mechanism of tRF-19-R118LOJX might be related to its function as an RNA silencer by targeting the S100A11 mRNA 3’UTR.

**Conclusion:**

tRFs would become novel diagnostic biomarkers and therapeutic targets of glioma, and the mechanism might be related to its post-transcriptionally regulation of gene expression by targeting mRNA 3’UTR.

**Supplementary Information:**

The online version contains supplementary material available at 10.1186/s12885-023-11532-8.

## Introduction

Gliomas are the most common primary malignant neuronal tumors, and glioblastoma (GBM) is the most dangerous one among them [1]. Although surgery combined with various comprehensive methods such as radiotherapy and chemotherapy has been performed for the therapy, the prognosis of GBM is still not optimistic. The five-year survival rate of GBM is approximately 6.8%, which is the lowest among all malignant brain tumors [2]. Therefore, exploring the molecular mechanism of GBM progression and finding more effective diagnostic markers and therapeutic targets have become urgent to rescue GBM patients.

In recent years, more and more scientific literature has shown that non-coding RNA (ncRNA) is an important part of the key regulators in the development of gliomas [3]. For example, long non-coding RNA PVT1 plays a role in the growth of glioma by regulating the miR-128-3p/GREM1 axis and BMP signaling pathway [4]. The circular RNA circNEIL3 has been confirmed to promote the occurrence and development of gliomas both in vivo and in vitro and has become a potential biological prognostic marker and therapeutic target for gliomas [5]. Other scholars found that the overexpression of small nucleolar RNA SNORD44 has inhibitory effects on the proliferation, apoptosis, and invasion of glioma cells [6]. The upstream region of rDNA, about 2 kb from the promoter, has transcriptional activity in glioma [7]. These results indicate that ncRNAs act an indispensable role in the pathogenesis of gliomas.

Transfer RNA (tRNA), a relatively small non-coding RNA, recognizes codons on mRNA and then transfers a particular amino acid to a growing polypeptide chain at the ribosomal site of protein synthesis during translation. Under hypoxia, sex hormone stimulation, and some other stressful conditions, tRNAs are cleaved to form small fragments of RNA (tRNA-derived Fragments, tRFs) and tRNA halves (tRNA Halves, tRHs), which are divided into 5’-tRHs, tRF-5, 3’-tRHs, tRF-3, i-tRF (internal tRFs), 5’U-tRFs, and tRF-1 through different cleavage positions of tRNAs [8]. tRF-5 is generated by the D-loop of mature tRNA cleaved by the Dicer enzyme, and tRF-3 is the 3’-end of tRNA, which is generated by the T-loop of mature tRNA cleaved by nuclease, angiopoietin, or Dicer enzyme. The i-tRF spans the anticodon loops. tRFs play important roles in many aspects such as protein translation [9], gene expression [10, 11] and cell cycle regulation [12], et al.

tRFs are also involved in the pathogenesis of cancer, central nervous system diseases, metabolic disorders, and other diseases. The functional mechanisms of tRFs have not been fully clarified. Several reports showed that tRFs are related to Argonaute proteins, and they could act as miRNAs by targeting mRNA 3’UTR to silence it [10, 13]. For example, tRF-T11 which is derived from the 5’ end of tRNAHis (GUG), can interact with AGO2 to directly target TRPA1 and suppress its expression through an RNA inference pathway in ovarian cancer cells [14]. Another study documented that tRFs derived from tRNAGlu, tRNAAsp, tRNAGly, and tRNATyr bind to YBX1 by competing with the 3’UTR of oncogene transcripts to inhibit the growth of breast cancer cells and reduce the invasion [15]. Meanwhile, 5’-tRFCys can promote oligomerization of an RNA-binding protein Nucleolin into a stabilizing higher-order transcript ribonucleoprotein complex that promotes metastatic lung colonization of breast cancer cells by enhancing cancer cell survival [16]. These studies of tRFs might provide new perspectives on the mechanisms of tumorigenesis.

So far, although accumulated research is currently focused on the role of dysregulated tRFs in the tumorigenesis of cancer, the expression and function of tRFs in glioma remain unclear. In this study, we screened the expression profiles of tRF subtypes in LGG and GBM, predicted the target genes of tRFs and their functions, identified the function of tRF-19-6SM83OJX, tRF-19-R118LOJX and one of tRF-19-R118LOJX’s target genes further, which will provide potential biomarkers and therapeutic targets for glioma.

## Materials and methods

### Retrieval of tRF expression data and potential target genes

In this study, TCGA-LGG/GBM datasets were analyzed to retrieve the tRF expression status using the MINTbase v2.0 dataset (https://cm.jefferson.edu/MINTbase). The expression information of tRFs was reported using the tRF’s normalized abundance (in RPM). The potential target genes of tRFs were explored using the tRF target database tRForest (https://trforest.com/) and tRFTar (http://www.rnanut.net/tRFTar/).

### Cell culture

Human glioma cell lines U87MG, U373MG, U251MG, and human astrocyte cell line (HA) SVG p12 were purchased from Shanghai Institutes for Biological Sciences (Shanghai, China). U87MG, U373MG and U251MG glioma cell lines were cultured in DMEM high glucose medium and SVG P12 cell line was cultured in MEM medium, supplemented with 10% fetal bovine serum (Gibco, NY, USA). The SV40-immortalized glial cell line SVG was derived from human fetal glial cells. This cell line is characterized as an astrocyte cell line that has been used for neurotoxicity experiments and in settings where human glial cells are relevant [17, 18]. During prolonged passage in vitro, SVG cells were found to change phenotype and karyotype. To ensure the consistency of the cell population used in the present study, we performed all experiments with the SVG p12 cell line that underwent 10–20 passages. All cells were incubated at 37 °C in an incubator (Forma Scientific, MA, USA) with a 5% CO_2_ concentration. The medium was updated every two days.

### RNA extraction and qPCR

Total RNA was extracted from HA and human U87, U251, and U373 glioma cell lines using Trizol reagent (Life Technologies, CA, USA), according to manufacturer instructions. Some RNA modifications were removed using rtStarTM tRF&tiRNA Pretreatment Kit (Cat# AS-FS-005, Arraystar). Using 2×miRNA P-RT Solution mix (Shanghai Sangon Biotech, China) and miRNA P-RT Enzyme mix (Shanghai Sangon Biotech, China), RNA was reversely transcribed into cDNA and tailed using poly-A-polymerase. TB Green Premix Ex Taq II (Takara, Japan) was used to identify the expression levels of the following four tRFs by 7500 Fast Real-time PCR (Applied Biosystems, CA, USA). The tRF sequences were obtained from the MINTbase v2.0 database and their specific primer sequences were designed as follows: tRF-19-R118LOJX: 5’-CCCAGTGCGCAATGGA-3’ (forward), tRF-19-6SM83OJX: 5’-CCGCGTGGCCTAATGGA-3’ (forward), tRF-30-87R8WP9N1EWJ: 5’-TCCCTGGTGTGTCTAGTGTGTTAG-3’ (forward), tRF-30-PNR8YP9LON4V: 5’-CATTGGTGTTCAGTGGTAGAATTCT-3’ (forward). The universal PCR Primer (RB661601, Sangon Biotech, Shanghai, China) was used as the reverse primer of the four tRFs. The program was set to preheat 30s at 95 °C, and then enter the cycle. Each cycle lasted 5s at 95 °C and then 34s at 60 °C. Repeat 40 cycles. The forward and reverse primers for internal reference gene U6 snRNA are available from Sangon Biotech (B661602, B661601, Shanghai, China). S100A11, the potential target mRNA for tRF-19-R118LOJX, was also verified by qRT-PCR and the mRNA transcript levels were normalized to those of the reference gene GAPDH. The specific primer sequences were designed as follows: S100A11, 5’-CCAGAAGTATGCTGGAAAGGATG-3’ (forward), 5’-CATCATGCGGTCAAGGACACCA-3’ (reward); GAPDH, 5’-GGACCTGACCTGCCGTCTAG-3’ (forward), 5’-TAGCCCAGGATGCCCTTGAG-3’ (reward). The relative quantitative 2^−ΔΔCt^ method was used to calculate the gene expression value.

### Functional enrichment analysis of the target genes of tRFs

The target genes of tRFs were subjected to Gene Ontology (GO) and Kyoto Encyclopedia of Genes and Genomes (KEGG) pathway enrichment analysis implemented by Metascape (https://metascape.org/) database. Only terms with a *P*-value < 0.01, a minimum count of 3, and an enrichment factor > 1.5 were collected and grouped into clusters based on their membership similarities. To visualize the membership matrix of genes involved in one group, a clustergram was displayed after enrichment analysis sorted by within the current cluster. The darkness of the orange color reflects the p-value of the given term. A functional analysis mapping gene to KEGG pathways was performed using the KEGG database (https://www.kegg.jp/) [19]. Gene set enrichment analysis (GSEA, https://www.omicshare.com/tools/) was performed to reveal the enrichment of signaling pathways and biological functions that were associated with the target genes of tRFs between LGG and GBM groups in the Chinese Glioma Genome Atlas (CGGA) database [20]. Normalized enrichment score (NES), nominal *P* value, and false discovery rate (FDR) *q* value were used for the statistical analysis. The cut-off criterion for GSEA was |NES|> 1, NOM *p* value < 0.05, and FDR *q* value < 0.25.

### Protein-protein interaction network and hub gene of the target genes of tRFs

To evaluate the association of the target genes of tRFs, the protein-protein interaction (PPI) enrichment analysis was performed by STRING (http://string-db.org/) with the minimum required interaction score = 0.4 and visualized by Cytoscape v.3.9.1. The hub genes were identified by Molecular Complex Detection plugin and the parameters were set at their default values.

### Cell transfection

tRF-19-R118LOJX and tRF-19-6SM83OJX inhibitors, siRNA targeted to S100A11 (si-S100A11) and their matching negative controls (NC) were designed and synthesized by GenePharma (Shanghai, China). The siRNA sequences targeting S100A11 were as follows: sense, 5’-GUGUCCUUGACCGCAUGAUTT-3’, and anti-sense, 5’-AUCAUGCGGUCAAGGACACTT-3’; si-NC sense, 5’-UUCUCCGAACGUGUCACGUTT-3’, and anti-sense, 5’-ACGUGACACGUU CGGAGAATT-3’. U251 and U87 cells were transfected with 0.2 μm tRF-19-R118LOJX or tRF-19-6SM83OJX inhibitor, si-S100A11 and their matching negative controls with Lipofectamine 3000 transfection reagent (Thermo Fisher Scientific, CA, USA) according to the manufacturer’s protocol. After 48 h of transfection, the cells were harvested for further experiments.

### Cell counting Kit-8 proliferation assay

Twenty-four hours after transfection, U251 and U87 cells were seeded in 96-well plates with a density of 3 × 10^4^ cells per well. After incubation for 24 h, 10 µl of CCK-8 reagent (Dojindo Molecular Technologies, Japan) was added to each well, and the cells were incubated at 37 °C for 4 h. The absorbance at 450 nm was detected by a microplate reader (Tecan, Switzerland).

### EdU assay

The cell proliferation was analyzed using BeyoClick™ EdU Kit with Alexa Fluor 488 (Beyotime, China). Twenty-four hours after transfection, U251 and U87 cells (1 × 10^4^ cells per well) were seeded in a 96-well plate for 24 h and then exposed to 50 µM EdU for 2 h at 37 °C. After fixed with 4% formaldehyde for 15 min, the cells were infiltrated with 0.3% Triton X-100 for 15 min. After washing, the cells were treated with 100 µl Click Additive Solution for 30 min and counterstained using Hoechst33342. The staining results were calculated under fluorescence microscope (IX-71, Olympus, Tokyo, Japan). Pictures of five fields of view randomly selected were taken to further analyze.

### TUNEL assay

The degrees of apoptosis in glioma cells were detected by TUNEL staining using a One Step TUNEL Apoptosis Assay Kit (Beyotime, China) according to the manufacturer’s instructions [21]. Briefly, at 2 days after transfection, the cells were fixed with 4% PFA and permeabilized with 0.3% Triton X-100, and then, the cells were incubated with the TUNEL reaction solution for 1 h at 37 °C in the dark. After rinsing with PBS, the cell nuclei were stained with Hoechst33342 for 5 min. Then, the stained cells were observed using a microscope (Olympus, Tokyo, Japan).

### Cell migration assays

Cell migration assay was conducted using a HoloMonitor M4 digital holographic cytometer from Phase Holographic Imaging (PHI, Lund, Sweden) according to the manufacturer’s protocols. Twenty-four hours after transfection, U251 and U87 cells were seeded into 6-well plates with a density of 3 × 10^5^ cells/ml. Twenty-four hours post seeding, the cells were tracked using the Hstudio and imaged every hour for 8 h. At the beginning of the analysis, five visually identifiable cells were selected for tracking in each group. The last image frame and the cell motility of each group were presented. Cell motility was showed in spatial X-Y plots.

### In vitro vasculogenic mimicry (VM) tube formation assay

The 96-well plate was coated with 100 µl Matrigel Basement Membrane Matrix (BD Biosciences, MA, USA) per well and then allowed to solidify for 30 min at 37 °C. The U251 and U87 cells transfected for 48 h were resuspended in 100 µl serum-free medium and seeded onto the surface of Matrigel at a density of 6 × 10^4^ cells per well. After incubation for 5 h, the cell vascular structures were observed under an inverted microscope (Olympus, Tokyo, Japan).

### Dual-luciferase reporter assay

The putative tRF-19-R118LOJX target binding sequence in the 3’UTR of S100A11 mRNA and its mutant sequence of the binding sites were amplified by PCR and cloned into pmirGLO Dual-luciferase miRNA Target Expression vector (Promega, Madison, WI, USA). HEK293T cells were seeded in a 96-well plate and co-transfected with the S100A11-3’UTR wild-type (Wt) or mutation type (Mut) pmirGLO vectors, and tRF-19-R118LOJX mimic or mimic NC (GenePharma, Shanghai, China) using Lipofectamine 3000 transfection Reagents. The luciferase activity was measured 48 h after transfection using a Dual-Luciferase Reporter Assay System (Promega, Madison, WI, USA), and the relative luminescence activity was normalized to the co-expressed Renilla.

### Western blot analysis

The cells were lysed with RIPA buffer (Beyotime Institute of Biotechnology, China) and the extracted proteins were transferred to the polyvinylidene fluoride membrane after separated by 12% SDS-PAGE. The membrane was blocked with 5% non-fat milk at room temperature for 2 h, and then incubated with the primary antibodies at 4 °C overnight, followed by incubation with the secondary antibodies. Immunoblots were visualized via Enhanced Development Chemiluminescence kit (Beyotime Institute of Biotechnology, China) and detected by a MicroChemi 4.2 Imaging System (DNR Bio-Imaging Systems, Israel). Band intensities were analyzed using ImageJ and were normalized to those of GAPDH. The primary antibodies against S100A11 and GAPDH were purchased from Proteintech.

### Statistical analysis

Each experiment was repeated three times. All differences were analyzed using GraphPad Prism 7 (GraphPad Software, CA, USA). The results were expressed as mean ± standard deviation. The differentiation between groups was compared with a two-tail unpaired Student’s t-test or one-way ANOVA. For the post hoc test, Tukey’s method was used to compare all possible group pairings, and Dunnett’s Method was used to compare treatment groups to a control group after one-way ANOVA. *P* < 0.05 was considered statistically significant.

## Results

### The profile of tRNA-derived fragments in glioma

**Fig. 1:**
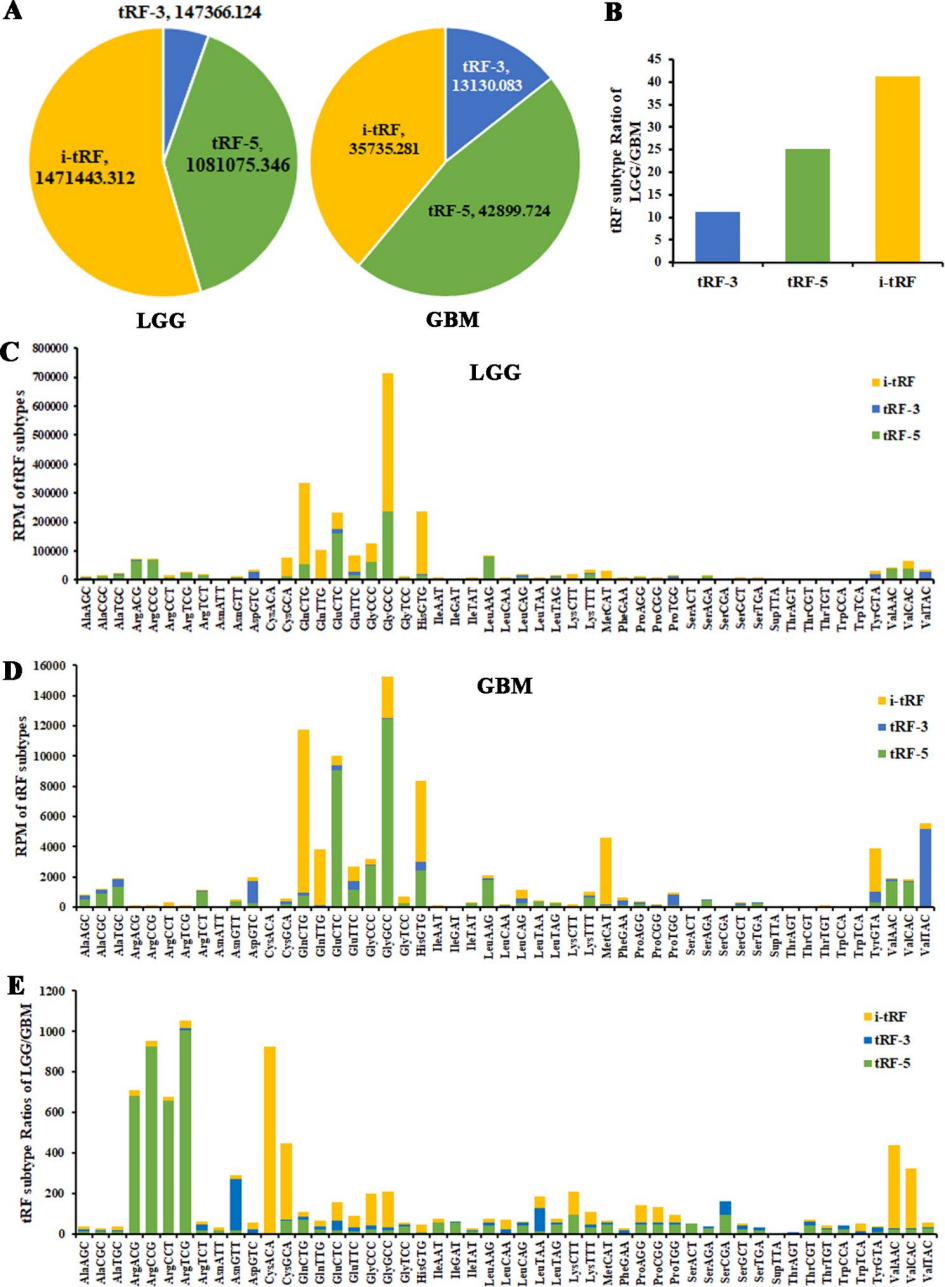
The proportions of tRF subtypes against tRNA isodecoders in the LGG and GBM groups regarding the abundance through MINTbase. The TCGA-LGG/GBM dataset analysis reveals the distributions (**A**) and proportions (**B**) of tRF subtypes in the LGG and GBM groups. Stacked plots for all tRF subtypes clustering by the anticodons of the tRNAs in the LGG (**C**) and GBM (**D**) groups. (**E**) Stacked plots for the proportions of tRF subtypes against tRNA isodecoders between the LGG and GBM groups

By analyzing TCGA-LGG/GBM dataset, the expression abundance of i-tRFs in the LGG group is the highest, accounting for 55%, followed by tRF-5s, accounting for 40%. The expression abundance of tRF-5s in the GBM group is the highest (47%), followed by i-tRFs (39%). tRF-3s account for the smallest proportion in both LGG and GBM groups (Fig. 1A). By calculating the expression abundance of the three tRF subtypes in LGG/GBM, it is confirmed that i-tRFs are downregulated most significantly in the GBM group (Fig. 1B). The tRF-2s derived from the decomposition of anticodon loops of tRNAs are included in i-tRFs. The tRF-1s derived from the 3’ ends of pre-tRNA are not included in the version 2.0 of MINTbase. By analyzing the expression abundance of all tRF subtypes against tRNA isodecoders in the LGG group, the highest one comes from tRFs derived from tRNA-GlyGCC, among which i-tRFs have the highest expression abundance, accounting for 66.96%. In GBM, tRFs with the highest expression abundance are also derived from tRNA-GlyGCC, and with the highest proportion of tRF-5s (81.89%) (Fig. 1C,D). The differentially expressed abundances of all tRF subtypes against tRNA isodecoders between LGG and GBM groups were analyzed. The greatest difference comes from tRFs derived from tRNA-ArgTCG. Other tRNA-Arg isoacceptors, such as tRNA-ArgACG/CCG/CCT, derived tRFs are also significantly down-regulated in the GBM group, and these tRFs are all tRF-5s. In addition, tRFs derived from tRNA-CysACA are also significantly down-regulated in the GBM group, mainly i-tRFs (Fig. 1E).

### The most abundant and differentially expressed tRFs in glioma

**Fig. 2:**
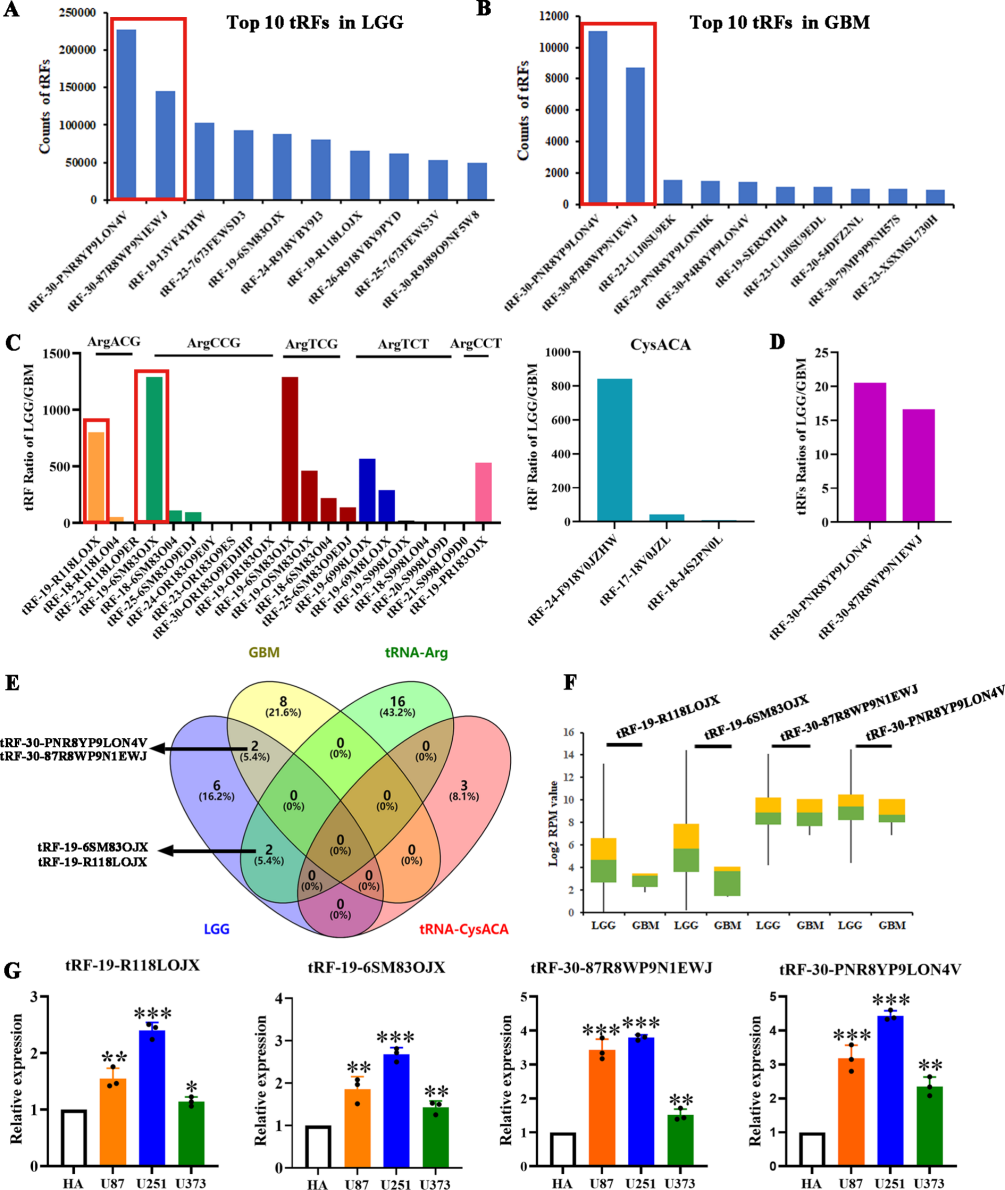
The abundance and differential expressed tRFs in LGG and GBM groups. The top 10 abundant tRFs in LGG (**A**) and GBM (**B**) groups. (**C**) The proportions of the tRFs derived from tRNA-ArgACG/CCG/TCG/TCT/CCT and tRNA-CysACA between LGG and GBM groups. (**D**) The ratio of the top two abundant tRFs between LGG and GBM groups. (**E**) Venn diagram formed using the number of the top 10 tRFs in LGG and GBM and tRFs derived from tRNA-ArgACG/CCG/TCG/TCT/CCT and tRNA-CysACA. (**F**) The expression of tRF-19-R118LOJX, tRF-19-6SM83OJX, tRF-30-87R8WP9N1EWJ and tRF-30-PNR8YP9LON4V in LGG and GBM groups. (**G**) The relative expressions of the four tRFs in U87, U251 and U373 glioma cells. Data represents mean ± SD (n = 3), *P < 0.05, **P < 0.01, ***P < 0.001, vs. HA group

After analysis, the top two abundant tRFs in LGG and GBM groups are tRF-30-PNR8YP9LON4V and tRF-30-87R8WP9N1EWJ, which belong to tRF-5s. The former is derived from tRNA-GluCTC, and the latter is derived from tRNA-GlyGCC/CCC (Fig. 2A, B). The LGG/GBM ratios of the most differentially expressed tRF subtypes derived from tRNA-Arg and tRNA-Cys in Fig. 1E were further analyzed, and the LGG/GBM ratios of tRF-30-PNR8YP9LON4V and tRF-30-87R8WP9N1EWJ, the top two tRFs in Fig. 2A and B, were calculated (Fig. 2C, D). Although the differential expressions of tRF-30-PNR8YP9LON4V and tRF-30-87R8WP9N1EWJ are obviously lower than those of tRNA-Arg or tRNA-Cys derived tRFs, their dysregulation in LGG and GBM tissues may still affect the expression of target genes or proteins due to their high expression abundance. Venn plot indicated that tRF-19-R118LOJX derived from tRNA-ArgACG and tRF-19-6SM83OJX derived from tRNA-ArgCCG coexist in the top10 tRFs in the LGG group (Fig. 2E). Figure 2 F further analyzed the expression changes of the most abundant or most differentially expressed four tRFs in the TCGA dataset between LGG and GBM groups. Except for tRF-30-87R8WP9N1EWJ, the expressions of other tRFs in the GBM group are obviously lower than those in the LGG group. The expressions of the four tRFs in U87, U251, and U373 glioma cell lines were analyzed by qPCR, all are significantly upregulated compared with the HA group, and the highest is found in U251 cells, as shown in Fig. 2G.

### The potential sites of tRF modifications and expressions of Dicer

**Fig. 3:**
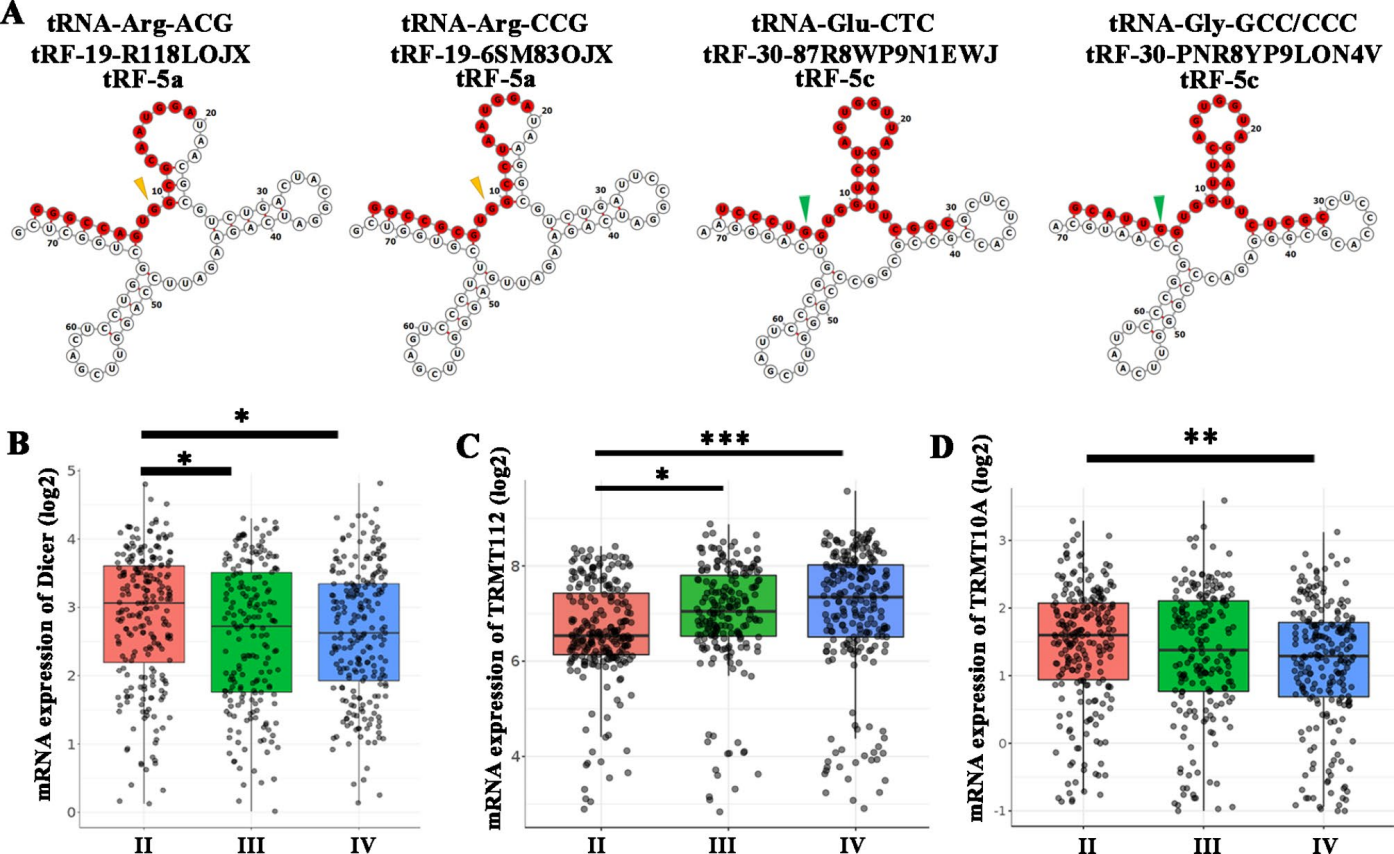
The four tRFs modification patterns and their potential enzymes. (**A**) Examples of tRNA structures of tRF-19-R118LOJX, tRF-19-6SM83OJX, tRF-30-87R8WP9N1EWJ and tRF-30-PNR8YP9LON4V were depicted. The red nucleotides indicate the four tRFs. The yellow and green arrowheads show the positions of m^1^G9 and m^2^G6 modifications, respectively. The relative mRNA expressions of Dicer (**B**), TRMT112 (**C**) and TRMT10A (**D**) in the II (n = 291), III (n = 334) and IV (n = 388) grades of glioma in CGGA dataset. Data represents mean ± SD, *P < 0.05, **P < 0.01, ***P < 0.001

By analyzing the secondary structure of tRFs, it was found that tRF-19-6SM83OJX and tRF-19-R118LOJX belong to tRF-5a, tRF-30-87R8WP9N1EWJ and tRF-30-PNR8YP9LON4V belong to tRF-5c. These derivatives may be generated by Dicer cleavage of tRNAs (Fig. 3A, Table 1). Figure 3B showed that the expression of Dicer in the GBM group was significantly lower than that in the grade II glioma group by analyzing the CGGA dataset. The m^2^G6 modification of tRNA-GlyGCC/CCC and m^1^G9 modification of tRNA-ArgACG/CCG may also affect the expression abundance of tRF-30-PNR8YP9LON4V, tRF-19-6SM83OJX and tRF-19-R118LOJX. By analyzing CGGA dataset, the expression of TRMT112 which modifies m^2^G6 was significantly higher in the GBM group than that in the grade II glioma group (Fig. 3C), while the expression of TRMT10A which modifies m^1^G9 in the GBM group was significantly lower than that in the grade II glioma group (Fig. 3D).

**Table 1:** The bioinformation of tRFs

tRF	Sequence	tRNA origin	Type	Length
tRF-19-R118LOJX	GGGCCAGT***G***GCGCAATGGA	ArgACG	tRF-5a	19
tRF-19-6SM83OJX	GGCCGCGT***G***GCCTAATGGA	ArgCCG	tRF-5a	19
tRF-30-87R8WP9N1EWJ	TCCCT***G***GTGGTCTAGTGGTTAGGATTCGGC	GluCTC	tRF-5c	30
tRF-30-PNR8YP9LON4V	GCATT***G***GTGGTTCAGTGGTAGAATTCTCGC	GlyGCC/ CCC	tRF-5c	30

The bold italic nucleotides indicate the potential sites of modification

### Function enrichment analysis of the target genes of tRFs

**Fig. 4:**
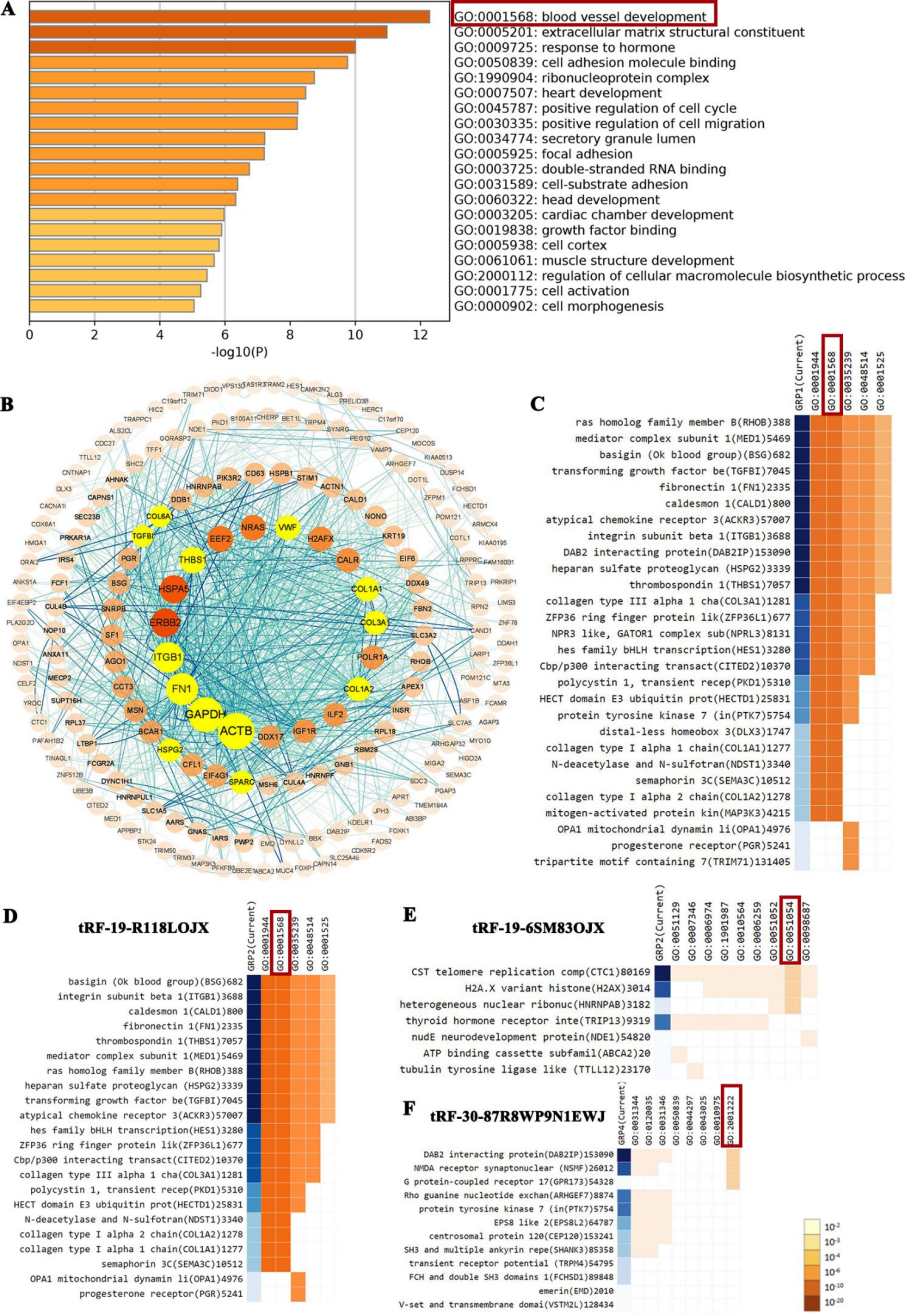
Function enrichment analysis of the target genes of tRF-19-R118LOJX, tRF-19-6SM83OJX, tRF-30-87R8WP9N1EWJ and tRF-30-PNR8YP9LON4V. (**A**) The target genes of top 20 modules were subjected to GO and KEGG enrichment, sorted by -log10 (P). (**B**) The PPI network of the hub genes (highlighted) with a higher degree of connectivity and enrichment analysis of the target genes of the four tRFs. Clustergram of the target genes of 4 tRFs enriched by GO: 0001568 (**C**); the target genes of tRF-19-R118LOJX, tRF-19-6SM83OJX and tRF-30-87R8WP9N1EWJ enriched by GO: 0001568, GO: 0051054 and GO: 2001222, respectively (**D-F**).

Target genes of tRF-19-6SM83OJX, tRF-19-R118LOJX, tRF-30-87R8WP9N1EWJ, and tRF-30-PNR8YP9LON4V were analyzed by tRForest and tRFTar database (see Table SI). Then, the functions enrichment analysis of target genes of the above four tRFs were obtained from Metascape. As presented in Fig. 4A, the function of differentially expressed tRFs’ target genes was mainly enriched in blood vessel development. The target protein interaction network was analyzed by STRING, and the hub genes were screened by Cytoscape using the Mcode plug-in (Fig. 4B). Figure 4 C showed the target genes of the above four tRFs mainly enriched in blood vessel development. Figure 4D-F indicated the target genes of tRF-19-R118LOJX enriched mostly in blood vessel development, the target gene of tRF-19-6SM83OJX enriched largely in positive regulation of DNA metabolic process, and the target genes of tRF-30-87R8WP9N1EWJ enriched mostly in the regulation of neuron migration. Due to the lack of sufficient target genes of tRF-30-PNR8YP9LON4V, no enrichment was obtained from the GO analysis. GSEA was further conducted to search the (KEGG) pathways and biological functions that were associated with the target genes of the above four tRFs. The focal adhesion pathway was activated. Moreover, the target genes of tRFs play an important role in the regulation of cell adhesion, cell development, and cell activation. The GSEA analysis also verified the GO analysis (Fig. S1).

### Elevated target genes of tRF-19-R118LOJX were associated with tumorigenesis and poor OS in glioma patients

**Fig. 5:**
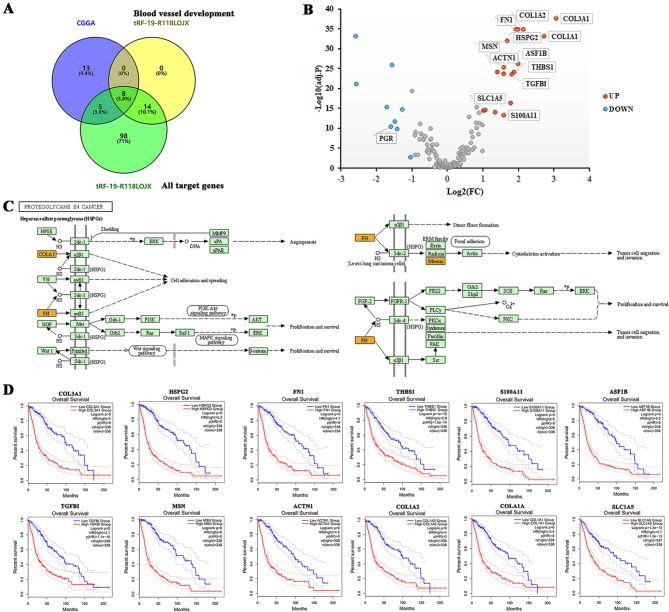
The upregulated target genes of tRF-19-R118LOJX promote tumorigenesis and predict poor overall survival of GBM patients. (**A**) Venn diagram for overlapped DEGs of CGGA between LGG and GBM, the target genes of tRF-19-R118LOJX enriched by blood vessel development and all target genes of tRF-19-R118LOJX. (**B**) Volcano plot of DEGs of CGGA and the differentially expressed target genes of tRF-19-R118LOJX (indicated using data tag) for LGG vs. GBM of CGGA. The red points indicate upregulated genes (n = 17), and the blue points indicate downregulated genes (n = 9) with absolute fold change values greater than 2 being statistically significant. Non-differentially expressed genes are indicated in gray. (**C**) KEGG mapping of proteoglycans in cancer is the most enriched pathway of the differentially expressed target genes of tRF-19-R118LOJX. Orange-marked nodes correspond to target genes from tRF-19-R118LOJX. (**D**) Overall survival analysis of the upregulated target genes of tRF-19-R118LOJX in GBM vs. LGG.

The Venn plot was used to select the genes crossed among the 26 genes differentially expressed more than 2-fold between LGG and GBM groups in the CGGA database, and all the target genes of tRF-19-R118LOJX, including the target genes enriched in blood vessel development. The results showed that 8 target genes of tRF-19-R118LOJX enriched in blood vessel development were differentially expressed between LGG and GBM groups (Fig. 5A). Figure 5B indicated all 13 differentially expressed target genes of tRF-19-R118LOJX between LGG and GBM groups in the CGGA database, among which 12 genes were significantly up-regulated and 1 gene was significantly down-regulated in the GBM group. KEGG pathway enrichment analysis of the target genes of tRF-19-R118LOJX validated proteoglycans in cancer were the highest matched object. tRF-19-R118LOJX might play a crucial role in angiogenesis, tumor cell migration, invasion, and proliferation, and survival through the target genes COLA1, FN1, and MSN (Fig. 5C). The survival analysis of GEPIA 2 showed that the 12 upregulated genes in the GBM group were positively correlated with the poor prognosis of patients, as shown in Fig. 5D.

### Elevated target genes of tRF-19-6SM83OJX were associated with poor OS in glioma patients

**Fig. 6:**
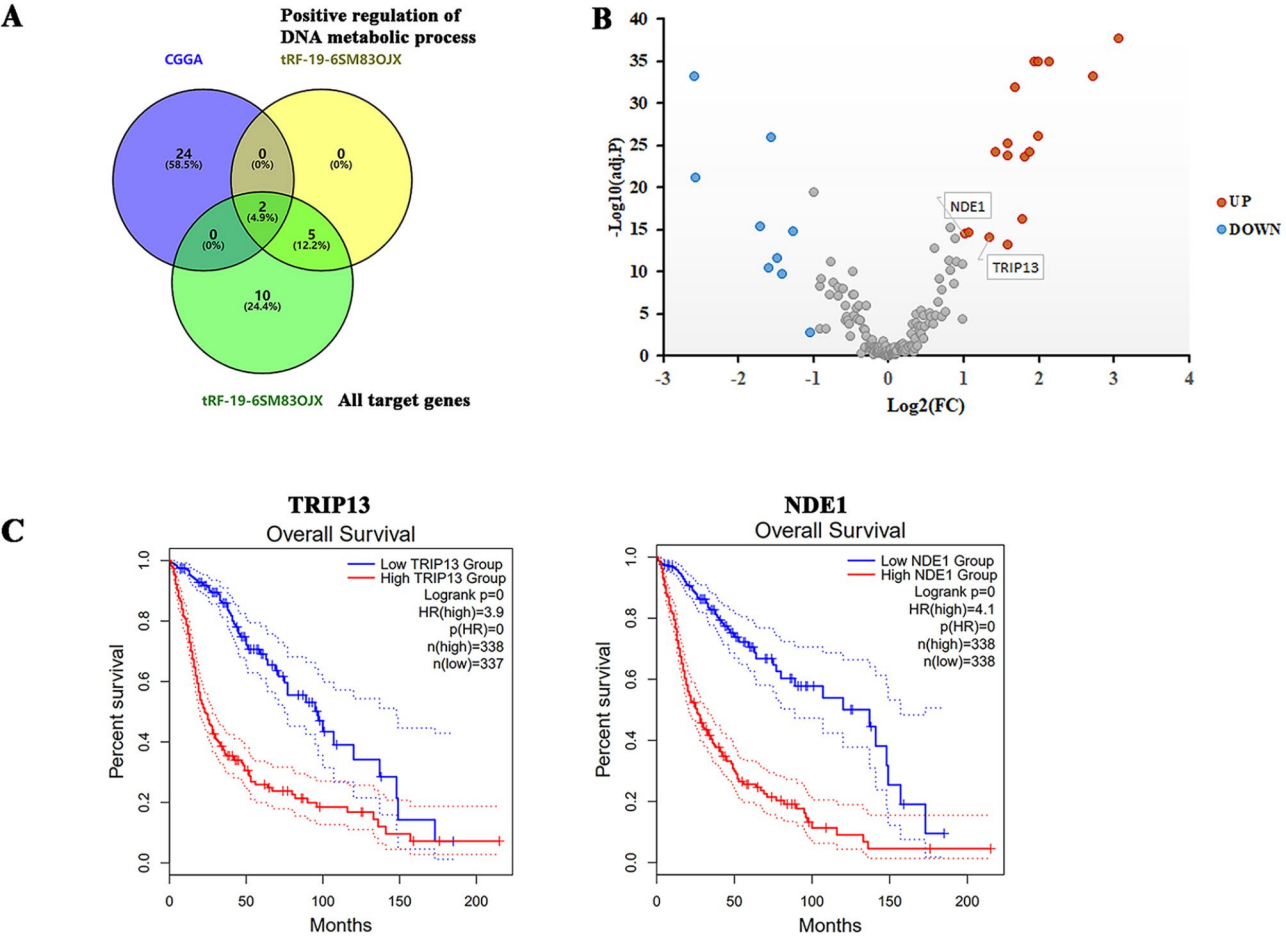
The upregulated target genes of tRF-19-6SM83OJX predict poor overall survival of GBM patients. (**A**) Venn diagram for overlapped DEGs of CGGA, the target genes of tRF-19-6SM83OJX enriched by positive regulation of DNA metabolic process, and all target genes of tRF-19-6SM83OJX. (**B**) Volcano plot of DEGs of CGGA and the differentially expressed target genes of tRF-19-6SM83OJX (indicated using data tag) for LGG vs. GBM of CGGA. The red points indicate upregulated genes (n = 2) with absolute fold change values greater than 2 being statistically significant. Non-differentially expressed genes are indicated in gray. (**C**) Overall survival analysis of the upregulated target genes of tRF-19-6SM83OJX in GBM vs. LGG.

Through the Venn plot, the genes intersected among the 26 genes differentially expressed more than 2-fold between LGG and GBM groups in the CGGA database, and all the target genes of tRF-19-6SM83OJX, including the target genes enriched to positive regulation of DNA metabolic process, were investigated. The results showed that the two target genes of tRF-19-6SM83OJX enriched in positive regulation of DNA metabolic process were differentially expressed between LGG and GBM groups (Fig. 6A). Figure 6B indicated the two target genes of tRF-19-6SM83OJX in the CGGA database were up-regulated in the GBM group. The survival analysis of GEPIA 2 verified that the two genes upregulated in the GBM group were positively correlated with the adverse prognosis of patients, as indicated in Fig. 6C. For the other two tRFs, no further analysis was performed. The expressions of tRF-30-87R8WP9N1EWJ showed no significant difference between LGG and GBM groups. For tRF-30-PNR8YP9LON4V, no enrichment was obtained from the GO analysis of its target genes.

### tRF-19-R118LOJX and tRF-19-6SM83OJX knockdown inhibit proliferation, migration and in vitro VM formation of glioma cells

**Fig. 7:**
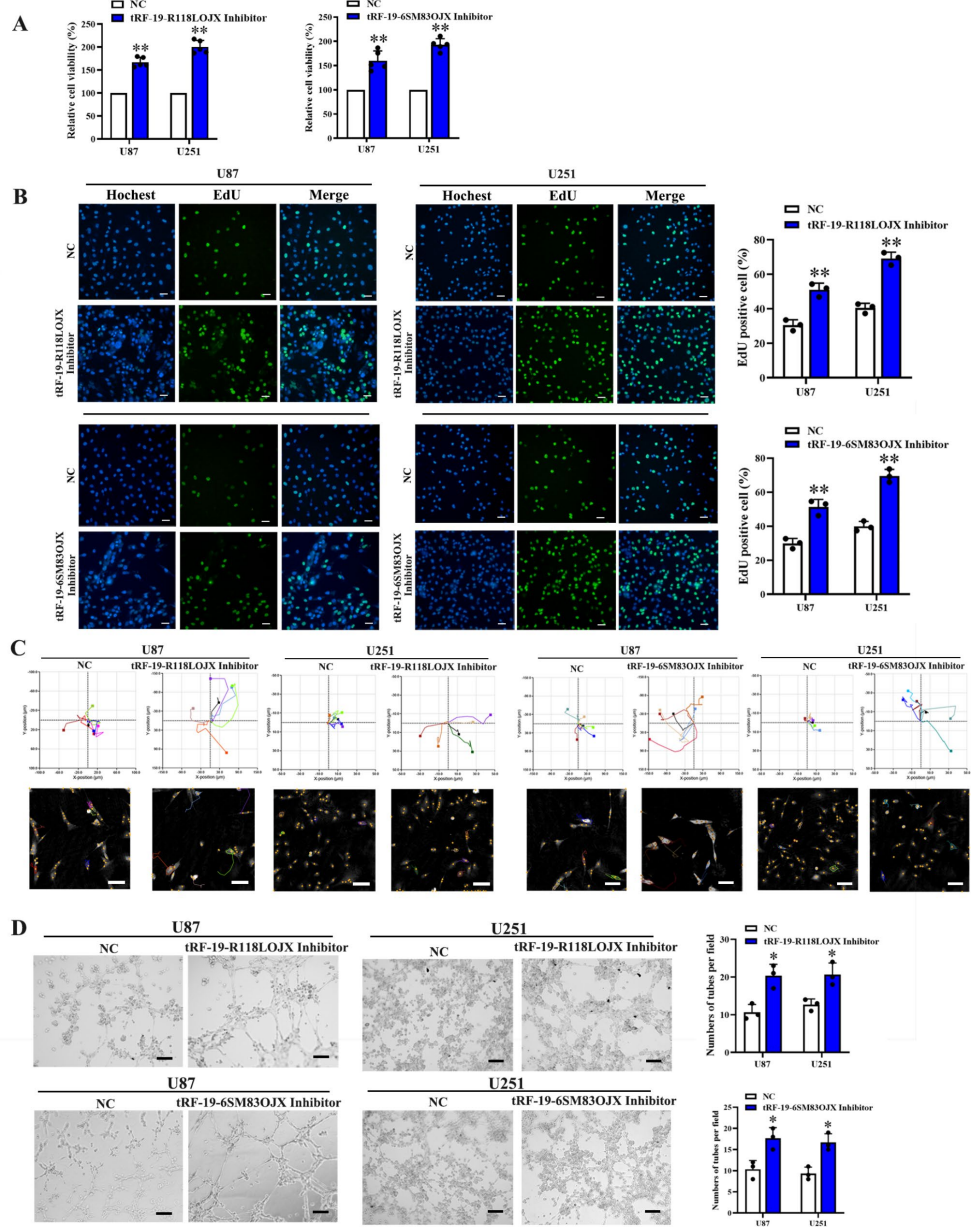
The tRF-19-R118LOJX and tRF-19-6SM83OJX inhibitors promote the proliferation, migration and in vitro VM formation of U87 and U251 glioma cells. The tRF-19-R118LOJX and tRF-19-6SM83OJX inhibitors increased the cell viability (n = 5) (**A**), cell proliferation rate (n = 3, Scale bars: 20 μm) (**B**), cell migration (n = 5, Scale bars: 100 μm) (**C**) and in vitro VM formation (n = 3, Scale bars: 200 μm) (**D**). Data represents mean ± SD, *P < 0.05, **P < 0.01, vs. NC group

To analyze the effect of dysregulated tRF-19-R118LOJX or tRF-19-6SM83OJX on malignant biological behaviors of U87 and U251 glioma cells, CCK-8 and EdU assays were performed to detect the changes of cell proliferation. The results showed that compared with inhibitor NC group, the cell viabilities and DNA synthesis in the tRF-19-R118LOJX or tRF-19-6SM83OJX inhibitor groups were significantly increased (Fig. 7A, B). Similarly, the results of cell migration and in vitro VM formation experiments showed that the cell migration distance and the number of tubes in the tRF-19-R118LOJX and tRF-19-6SM83OJX inhibitor groups were significantly increased compared with the inhibitor NC group (Fig. 7C, D).

### tRF-19-R118LOJX affected glioma cell proliferation, migration and in vitro VM formation via negatively regulating the S100A11 expression

**Fig. 8:**
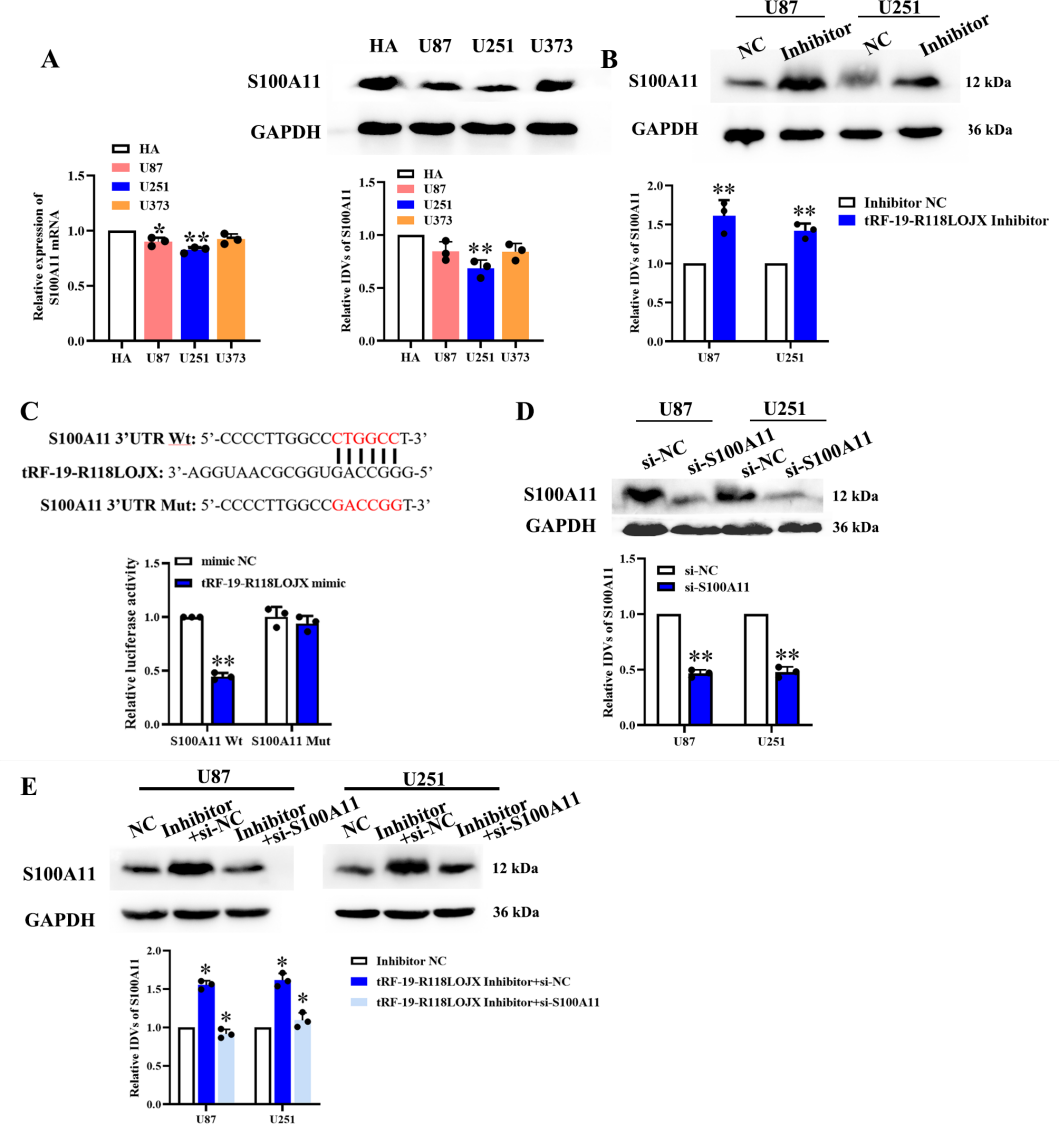
tRF-19-R118LOJX downregulates the S100A11 expression by directly targeting its 3’UTR. (**A**) The mRNA and protein expressions of S100A11 were detected using qPCR and western blot assay in HA, U251, U87 and U373 cells. Data represents mean ± SD (n = 3), *P < 0.05, **P < 0.01 vs. HA group. (**B**) The protein expressions of S100A11 were detected by western blot assays in U251 and U87 cells treated with tRF-19-R118LOJX inhibitor (n = 3). (**C**) The luciferase activity of Fluc/Rluc was detected by dual-luciferase reporter assays (n = 5). (**D**) After si-S100A11 induced mRNA knockdown, the S100A11 protein expressions in U251 and U87 cells were determined by western blot assays (n = 3). (**E**) The protein expressions of S100A11 were detected by western blot assays in U251 and U87 cells treated with tRF-19-R118LOJX inhibitor alone or in combined with si-S100A11 (n = 3). Data represents mean ± SD, *P < 0.05, **P < 0.01 vs. NC group. All the blots were cropped prior to hybridization with primary antibodies. The original blots are presented in Fig. S3-S6.

**Fig. 9:**
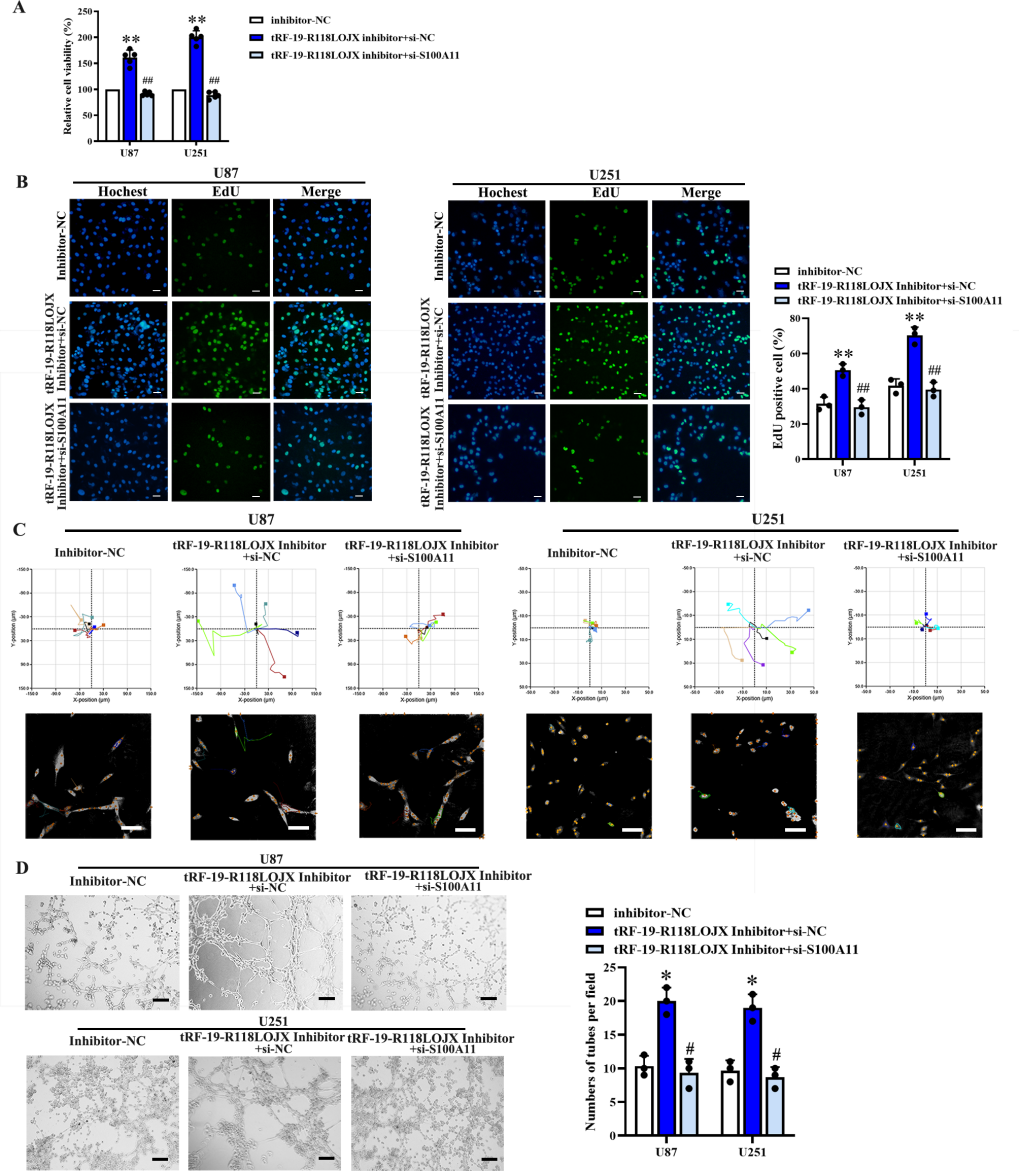
The tRF-19-R118LOJX inhibitor promotes the proliferation, migration and in vitro VM formation of U87 and U251 glioma cells via S100A11. The knockdown of S100A11 reversed the cell viability (n = 5) (**A**), cell proliferation rate (n = 3, Scale bars: 20 μm) (**B**), cell migration (n = 5, Scale bars: 100 μm) (**C**) and in vitro VM formation (n = 3, Scale bars: 200 μm) (**D**) promoted by tRF-19-R118LOJX inhibitor. Data represents mean ± SD, *P < 0.05, **P < 0.01 vs. Inhibitor-NC group; ^#^*P* < 0.05, ^##^*P* < 0.01 vs. tRF-19-R118LOJX inhibitor + si-NC group

tRF-19-R118LOJX was predicted to target the 3’UTR of S100A11 mRNA by tRFTar database. In HA, U251, U87 and U373 cells, qPCR and western blot experiments showed the mRNA and protein expressions of S100A11 are significantly lower in U251 cells than those in HA cells, which were opposite to the tRF-19-R118LOJX expressions in the above cell lines (Fig. 8A). Western blot experiments confirmed that the protein expressions of S100A11 were significantly up-regulated in the tRF-19-R118LOJX inhibitor group compared to the inhibitor NC group (Fig. 8B). Dual-luciferase reporter assay was employed to determine whether tRF-19-R118LOJX targets S100A11 directly. The results indicated that tRF-19-R118LOJX mimic significantly decreased the luciferase activity of the S100A11-3’UTR-Wt group, while no significant change of the luciferase activity of the S100A11-3’UTR-Mut group was observed (Fig. 8C). Then, S100A11 mRNA was knocked down using siRNA in the U87 and U251 cells and validated by western blot assay (Fig. 8D). To verify whether the targeted binding of tRF-19-R118LOJX with S100A11 could affect the glioma cell proliferation, migration and in vitro VM formation, the expression of S100A11 was knocked down in the tRF-19-R118LOJX inhibitor group (Fig. 8E). Compared with the treatment of tRF-19-R118LOJX inhibitor alone, the knockdown of S100A11 combined with the tRF-19-R118LOJX inhibitor reversed the increased cell proliferation (Fig. 9A, B), decreased cell apoptosis (Fig. S2), migration (Fig. 9C) and in vitro VM formation (Fig. 9D) induced by tRF-19-R118LOJX inhibitor.

## Discussion

Glioma is one of the most aggressive tumors with the highest death rate among all primary brain malignancies. The ncRNAs, such as long ncRNAs, microRNA, and circular RNAs are commonly dysregulated in GBM and play crucial roles in many biological processes connected with glioma initiation and progression. Recently, through the high throughput second-generation sequencing techniques, a novel group of ncRNAs, derived from tRNAs, has been revealed. The abnormal expression of tRFs in various cancers can not only be used as a marker for diagnosis and prognosis but also have the potential as a new target for treatment [22, 23].

To explore the abnormal expression of tRFs in glioma tissues, we analyzed the expression abundance of tRFs in LGG and GBM groups in the MINTbase v2.0 database. tRFs were mainly i-tRFs in the LGG group and tRF-5s in the GBM group. The LGG/GBM ratio of the expression abundance of tRFs suggested that compared with the LGG group, the expression abundance of i-tRFs in the GBM group was down-regulated, while the expression abundance of tRF-5s was up-regulated. The detail of tRFs biogenesis remains a matter of debate. Studies have shown that the generation of tRF-5s and tRF-3s is Dicer-dependent [24, 25]. Other studies have shown that Dicer does not affect the generation of the two [11, 26]. Di Fazio A et al. confirmed recently that the main reason for the differences in Dicer dependence is the different sample sources or lineages [27]. Dicer depletion leads to instability of pre-tRNA, tRNA 3’-end, and mature tRNA, significantly reducing 18-22nt long tRNA derivatives. The way i-tRFs are generated is not yet clear. The expressions of tRFs derived from tRNA-GlyGCC were the highest in LGG and GBM groups, and i-tRFs were predominant in the LGG group, and tRF-5s were predominant in the GBM group, suggesting that the pathological grade of glioma may affect the tRNA cleavage and the generation type of derivatives. The high expression of tRFs derived from tRNA-GlyGCC is not only found in glioma tissues, but also in other cancer tissues such as ovarian cancer and nasopharyngeal carcinoma [28, 29], human embryonic stem cell [30], or some physiological or pathological conditions [31, 32]. By analyzing the LGG/GBM ratio of tRFs expression abundance, it was found that the most down-regulated tRFs in the GBM group were derived from tRNA-ArgTCG/ACG/CCG (belonging to tRF-5s), tRNA-ArgCCT and tRNA-CysACA (belonging to i-tRFs), which partially echoed the report by Di Fazio A et al., where the authors showed that the incubation with Dicer led to the reduction of tRNA-Arg levels.

In the GBM group, tRF-19-R118LOJX, tRF-19-6SM83OJX, and tRF-30-PNR8YP9LON4V all belong to tRF-5s, derived from tRNA-ArgACG, tRNA-ArgCCG, and tRNA-GlyGCC/CCC, respectively, which can be recognized by Dicer [27, 33]. We speculated that the reason for the downregulation of the above tRFs may be related to the expressions of Dicer in different grades of glioma tissues. By analyzing Dicer’s expression changes in different grades of glioma in the CGGA database, it was found that the changing trend was consistent with that of tRF-5s such as tRF-19-R118LOJX, suggesting that the down-regulated Dicer in the GBM group might be one of the reasons for reducing biogenesis of tRF-5s. On the other hand, the downregulation of tRFs may be related to the modification levels of tRNAs. The modification of tRNAs is important for correct folding and structural stability, affecting the biogenesis of tRFs. Methylation is the most frequent post-transcriptional tRNA modification. Studies have confirmed that the 5’-terminal of tRNA-GlyGCC/CCC has a low level of m^2^G6 modification [34]. Recent studies have confirmed that the modification enzyme in m^2^G6 is THUMPD3. However, THUMPD3 alone could not catalyze tRNA methylation independently. The activation of the tRNA methyltransferase of THUMPD3 needs the formation of a complex with TRMT112, a universal activator for both RNA and protein methyltransferases [35]. By analyzing the CGGA dataset, the expression of THUMPD3 in the GBM group was not significantly changed compared with that in the LGG group, while the expression of TRMT112 in the GBM group was significantly increased compared with that in the grade II glioma group. The highly expressed TRMT112 in the GBM group may promote the formation of the THUMPD3-TRMT112 complex, and increase the methylation modification of tRNA m^2^G6, thereby increasing the stability of tRNA and reducing the biogenesis of tRF-5s. Similarly, the m^1^G9 modification existed in the tRF-5s derived from tRNA-ArgACG/CCG [33, 36]. TRMT10A is m^1^G9-specific tRNA methyltransferase. Vilardo E et al. confirmed that upon knockout of TRMT10A, the modification of G9 to m^1^G was abolished and the steady-state levels of tRNA-iMetCAT were decreased [37]. By analyzing the CGGA dataset, the expression of TRMT10A in the GBM group was significantly lower than that in the grade II glioma group, which was consistent with the downward trend of the above tRF-5s, suggesting that the m^1^G9 modification level of tRNA-ArgACG/CCG in the GBM group might not be the main factor affecting the stability of the above tRF-5s.

Yang WQ et al. verified that knocking out THUMPD3 hampered cell proliferation and global protein synthesis in HEK293T cells [35]. Similarly, Dai Z et al. and Orellana EA et al. confirmed that METTL1 reduced the expression of m^7^G-modified tRNAs, such as LysCTT/TTT and Arg-TCT-4-1, and global mRNA translation [38, 39]. The above results suggest that the up-regulated TRMT112 in the GBM group may increase the stability of tRNA-GlyGCC/CCC through the THUMPD3-TRMT112 complex, promote the translation efficiency of mRNA enriched in GGC/GGG codon, decoded by tRNA-GlyGCC/CCC. We analyzed the GGC/GGG codon frequency in the top10 up-regulated genes between the LGG and GBM groups in the CGGA dataset. The frequency of the GGC/GGG codon was significantly higher than that of the CGT/CGG codon decoded by tRNA-ArgACG/CCG (Table SII), suggesting that the tRNA-GlyGCC/CCC in the GBM group may upregulate protein expression by promoting mRNA translation efficiency.

Abnormally expressed tRFs not only regulate the malignant biological behavior of tumor cells, but also affect the growth of tumor vessels. To explore the role of the four tRFs with high and differential expressions in LGG and GBM in the development of glioma, we performed a functional enrichment analysis of the target genes of the four tRFs. The function of 28 target genes was related to blood vessel development, of which 22 genes were target genes of tRF-19-R118 LOJX, and the hub genes included TGFBI, ITGB1, THBS1, etc. In addition to the target genes of tRF-19-R118 LOJX, whether some pro-angiogenic factors such as VEGF, FGF, HIF-1α are involved in the blood vessel development induced by tRF-19-R118LOJX is also worth exploring in the future. Similarly, KEGG mapping of the target genes of tRF-19-R118LOJX confirmed that tRF-19-R118LOJX may play a critical role in angiogenesis, tumor cell migration, invasion, and proliferation. The above analysis results suggested that the expression change of tRF-19-R118LOJX may regulate the angiogenesis of glioma. Similar to our findings, Zhu XL et al. showed in the rat common carotid artery intimal hyperplasia model, the target gene of tRNA-GlnCTG-derived fragments played a crucial role in the regulation of vascular biological behavior [32]. Since the expression of tRF-30-87R8WP9N1EWJ in the GBM group had no significant change compared with that in the LGG group, and tRF-30-PNR8YP9LON4V did not find the binding target gene, we analyzed the differential expression of the target genes of tRF-19-R118LOJX and tRF-19-6SM83OJX in the CGGA dataset. The expressions of 12 tRF-19-R118LOJX target genes and 2 tRF-19-6SM83OJX target genes in the GBM group were positively correlated with the poor prognosis of patients, suggesting that differentially expressed tRFs in different grades of glioma tissues may affect the occurrence and prognosis of cancer. Further, the function of tRF-19-R118LOJX was identified in U87 and U251 glioma cells, which could inhibit the cell proliferation, migration and in vitro VM formation. Growing evidence suggests that tRFs act as the RNA silencer via targeting mRNA 3’UTR and implicate in the pathogenesis of cancers [10, 13, 14]. Dysregulation of protein kinases is implicated in various processes of carcinogenesis. Many protein kinase inhibitors had been approved by FDA because of several landmark clinical trials. Overexpression of tRiMetF31 profoundly suppressed migration and angiogenesis of breast cancer cells by silencing PFKFB3, which might represent a target molecule for therapeutic intervention [40, 41]. Among the 12 tRF-19-R118LOJX target genes, S100A11 showed the highest potential to bind via the 3’UTR. Dual-luciferase reporter assays were conducted to confirm the binding site of tRF-19-R118LOJX on the 3’UTR of S100A11 mRNA. Importantly, the protein expressions of S100A11 were significantly upregulated induced by tRF-19-R118LOJX inhibitor. The tRF-19-R118LOJX affected the glioma cell proliferation, apoptosis, migration and in vitro VM formation via targeted binding and negative regulation of S100A11. Members of S100 family proteins are known to play critical roles in cancer progression and angiogenesis [42, 43].

In human hepatocellular carcinoma, EIF3C activated expression of S100A11 involved in EIF3C-exosome increased tube formation in angiogenesis. [44] S100A11 also plays an essential role in glioma. Overexpression of S100A11 promoted GBM cell growth, epithelial-mesenchymal transition, migration, invasion, and generation of glioma stem cells. [45] Therefore, our results suggested that tRF-19-R118LOJX could function as a tumor suppressor, and the mechanism might be related to its post-transcriptionally regulation of gene expression by targeting mRNA 3’UTR. The expression and role of tRFs in patients with different grades of glioma and glioma cells have not been reported yet. Further experiments were necessary to identify more targets and functions of tRF-19-R118LOJX, tRF-19-6SM83OJX, and tRF-30-PNR8YP9LON4V in gliomas.

## Conclusion

In conclusion, the current study analyzed the dysregulated tRFs in LGG and GBM and their potential target genes. tRF-19-R118LOJX could act as a tumor suppressor in glioma cells and S100A11 was a direct target of tRF-19-R118LOJX in regulating glioma development. These finding suggest that tRFs may be associated with GBM pathogenesis and act as potential biomarkers and therapeutic targets for GBM.

### Electronic supplementary material

Below is the link to the electronic supplementary material.

Supplementary Material 1

Supplementary Material 2

## Data Availability

The datasets used and/or analyzed during the current study are available from the corresponding author on reasonable request.
