# Supplementary material for: Expression profiles and function prediction of tRNA-derived fragments in glioma
Source: BMC Cancer. 2023 Oct 20;23:1015. doi: 10.1186/s12885-023-11532-8 (PMC10588164; doi:10.1186/s12885-023-11532-8)
Supplement: Supplementary file 1 — Supplementary Material 1 [file 12885_2023_11532_MOESM1_ESM.docx]

Supplemental Table I The potential target genes of tRFs

| tRF-19-R118LOJX | tRF-19-6SM83OJX | tRF-30-87R8WP9N1EWJ | tRF-30-PNR8YP9LON4V |
| --- | --- | --- | --- |
| HES1 | NDE1 | LYRM4 | GORASP2 |
| EIF4EBP2 | BBX | C17orf103 | PLEKHG4 |
| EEF2 | CTC1 | PTK7 | TRIM71 |
| FN1 | ABCA2 | PWP2 | MECP2 |
| OPA1 | DDX17 | CACNA1I | PLA2G2D |
| ANKRD52 | GFOD1 | COX6A1 | FCGR2A |
| APPBP2 | IRS4 | FOXK1 | CNTNAP1 |
| PRKAR1A | SF1 | AMZ1 |  |
| DGKD | TTLL12 | PIANP |  |
| RPN2 | MRPL52 | COX6A1P2 |  |
| AHNAK | TRIP13 | ALS2CL |  |
| COL1A2 | ANKRD54 | ZNF76 |  |
| DYNC1H1 | H2AFX | TFCP2L1 |  |
| MSH6 | HNRNPAB | STIM1 |  |
| NAV2 | MFSD5 | EMD |  |
| RHOB | RPL18 | CPT1C |  |
| ZNF512B | YRDC | MYLK2 |  |
| ABI3BP |  | CCDC159 |  |
| BCAR1 |  | GPR173 |  |
| COL6A1 |  | PRKRIP1 |  |
| ERBB2 |  | FCHSD1 |  |
| FAM160B1 |  | PGAP3 |  |
| FBN2 |  | PAFAH1B2 |  |
| FBXO31 |  | VSIG10L |  |
| FOXP1 |  | DAB2IP |  |
| HECTD1 |  | CDK5R2 |  |
| HERC1 |  | KIAA0195 |  |
| LTBP1 |  | TMEM184A |  |
| MED1 |  | ZNF282 |  |
| SEC23B |  | CAMK2N2 |  |
| SLC39A14 |  | LMX1B |  |
| SPARC |  | ANXA11 |  |
| STK24 |  | ZNF286A |  |
| SUPT16H |  | TBC1D26 |  |
| SYNRG |  | RFTN1 |  |
| TFF1 |  | AC002472.13 |  |
| AARS |  | PEX26 |  |
| ACKR3 |  | ARHGEF7 |  |
| ACTN1 |  | COTL1 |  |
| AGO1 |  | SLC25A48 |  |
| ANKS1A |  | IL17REL |  |
| CALD1 |  | JPH3 |  |
| CAND1 |  | ZFPM1 |  |
| CDC27 |  | MTA3 |  |
| CHERP |  | CEP120 |  |
| CITED2 |  | DNAH10OS |  |
| COL3A1 |  | LRRC56 |  |
| CRIM1 |  | POLR1A |  |
| CUL4A |  | LIMS3 |  |
| CUL4B |  | BET1L |  |
| DDAH1 |  | GPR114 |  |
| DDB1 |  | UBE3B |  |
| DYNLL2 |  | SHANK3 |  |
| EIF4G1 |  | PFKFB3 |  |
| FCF1 |  | MUC4 |  |
| GNB1 |  | ORAI2 |  |
| HSPA5 |  | C17orf70 |  |
| HSPG2 |  | FADS2 |  |
| IARS |  | NSMF |  |
| IGF1R |  | DLX3 |  |
| INSR |  | EMID1 |  |
| ITGB1 |  | SH3TC1 |  |
| KRT19 |  | CELF2 |  |
| KRT80 |  | EPS8L2 |  |
| LARP1 |  | RBM28 |  |
| LRPPRC |  | SLC29A4 |  |
| MIGA2 |  | FCAMR |  |
| MSN |  | AGAP3 |  |
| NAV3 |  | MEF2BNB |  |
| NDST1 |  | TRPM4 |  |
| NONO |  | AC010642.1 |  |
| NRAS |  | POM121 |  |
| NUCKS1 |  | PDXK |  |
| PEG10 |  | VSTM2L |  |
| PGR |  | DIDO1 |  |
| PKD1 |  | TAS1R3 |  |
| SEMA3C |  | NPRL3 |  |
| TGFBI |  | CAPN14 |  |
| THBS1 |  | SHC2 |  |
| TINAGL1 |  | C19orf12 |  |
| TRAM2 |  | DDX49 |  |
| TRIM37 |  | TNRC18 |  |
| VAMP3 |  | DOT1L |  |
| VPS13D |  | ARHGAP32 |  |
| VWF |  | SDC3 |  |
| ZDHHC8 |  | TRIM50 |  |
| ZFP36L1 |  | ARMCX4 |  |
| ACTB |  | HIC2 |  |
| S100A11 |  | RP5-966M1.6 |  |
| SLC1A5 |  | KIAA0513 |  |
| GAPDH |  | CHRNB4 |  |
| NOP10 |  | POM121C |  |
| RPL37 |  | MYO1G |  |
| ALG3 |  | MUM1 |  |
| CALR |  | MAP3K3 |  |
| CAPNS1 |  | BTBD9 |  |
| CFL1 |  | PLEKHA4 |  |
| GNAS |  |  |  |
| HMGA1 |  |  |  |
| HNRNPUL1 |  |  |  |
| HSPB1 |  |  |  |
| TRAPPC1 |  |  |  |
| APEX1 |  |  |  |
| APRT |  |  |  |
| ASF1B |  |  |  |
| BAMBI |  |  |  |
| BSG |  |  |  |
| CCT3 |  |  |  |
| CD63 |  |  |  |
| DUSP14 |  |  |  |
| EIF6 |  |  |  |
| HIGD2A |  |  |  |
| HNRNPF |  |  |  |
| KDELR1 |  |  |  |
| MOCOS |  |  |  |
| PIK3R2 |  |  |  |
| PRELID3B |  |  |  |
| SLC3A2 |  |  |  |
| SLC7A5 |  |  |  |
| SNF8 |  |  |  |
| SNRPB |  |  |  |
| UBE2E1 |  |  |  |
| YRDC |  |  |  |
| COL1A1 |  |  |  |
| ILF2 |  |  |  |

Supplemental Table II The frequency and numbers of m^1^G9-modified tRNA-ArgACG/CCG-dependent codens and m^2^G6-modified tRNA-GlyGCC/CCC-dependent codens in the top 10 differentially expressed genes between GBM and LGG.

| tRF target genes | tRNA-Arg | | tRNA-Gly | |
| --- | --- | --- | --- | --- |
|  | CGT | CGG | GGC | GGG |
| COL3A1 | 7.5/11 | 3.4/5 | 40.9/60 | 21.8/32 |
| COL1A1 | 4.85 | 19.8/29 | 2.7/4 | 86/126 |
| COL1A2 | 14.6/20 | 4.4/6 | 62.9/86 | 12.4/17 |
| ASF1B | 4.9/1 | 14.8/3 | 39.4/8 | 9.8/2 |
| HSPG2 | 7.3/32 | 17.1/75 | 50.1/220 | 27.5/121 |
| FN1 | 5.7/14 | 4.9/12 | 23.7/58 | 15.1/37 |
| THBS1 | 5.7/14 | 4.9/12 | 23.7/58 | 15.1/37 |
| TGFBI | 2.9/2 | 14.6/10 | 27.8/19 | 20.5/14 |
| PLEKHA4 | 8.6/5 | 22.3/13 | 30.8/18 | 27.4/16 |
| MSN | 20.8/12 | 19/11 | 15.6/9 | 6.9/4 |

The values in the table body present frequency per thousand/number of codens in the coding sequence of tRF target gene.
