# Supplementary material for: Expression profiles and function prediction of tRNA-derived fragments in glioma
Source: BMC Cancer. 2023 Oct 20;23:1015. doi: 10.1186/s12885-023-11532-8 (PMC10588164; doi:10.1186/s12885-023-11532-8)
Supplement: Supplementary file 2 — Supplementary Material 2 [file 12885_2023_11532_MOESM2_ESM.docx]

**Supplementary Figures**

**Expression profiles and function prediction of tRNA-derived fragments in glioma**

**This file includes:**

**Fig. S1** GSEA plots showed the (KEGG) pathways and biological functions that were enriched in the target genes of tRF-19-R118LOJX, tRF-19-6SM83OJX, tRF-30-87R8WP9N1EWJ, and tRF-30-PNR8YP9LON4V.

**Fig. S2** The tRF-19-R118LOJX inhibitor inhibits apoptosis of U87 and U251 glioma cells via S100A11.

**Fig. S3** The original images of the western blot in Figure 8A.

**Fig. S4** The original images of the western blot in Figure 8B.

**Fig. S5** The original images of the western blot in Figure 8D.

**Fig. S6** The original images of the western blot in Figure 8E.

**
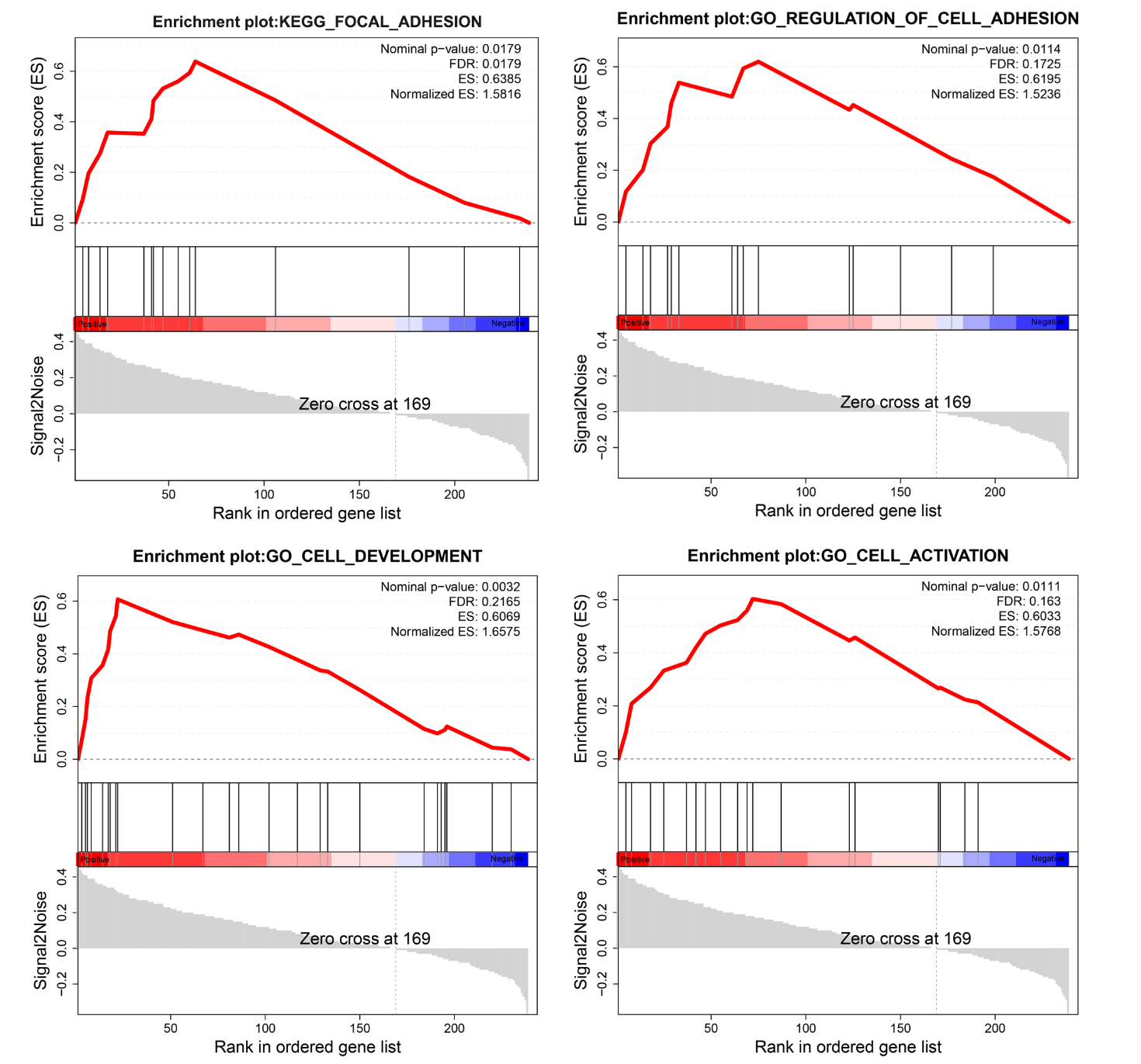
Fig. S1** GSEA plots showed the (KEGG) pathways and biological functions that were enriched in the target genes of tRF-19-R118LOJX, tRF-19-6SM83OJX, tRF-30-87R8WP9N1EWJ, and tRF-30-PNR8YP9LON4V.


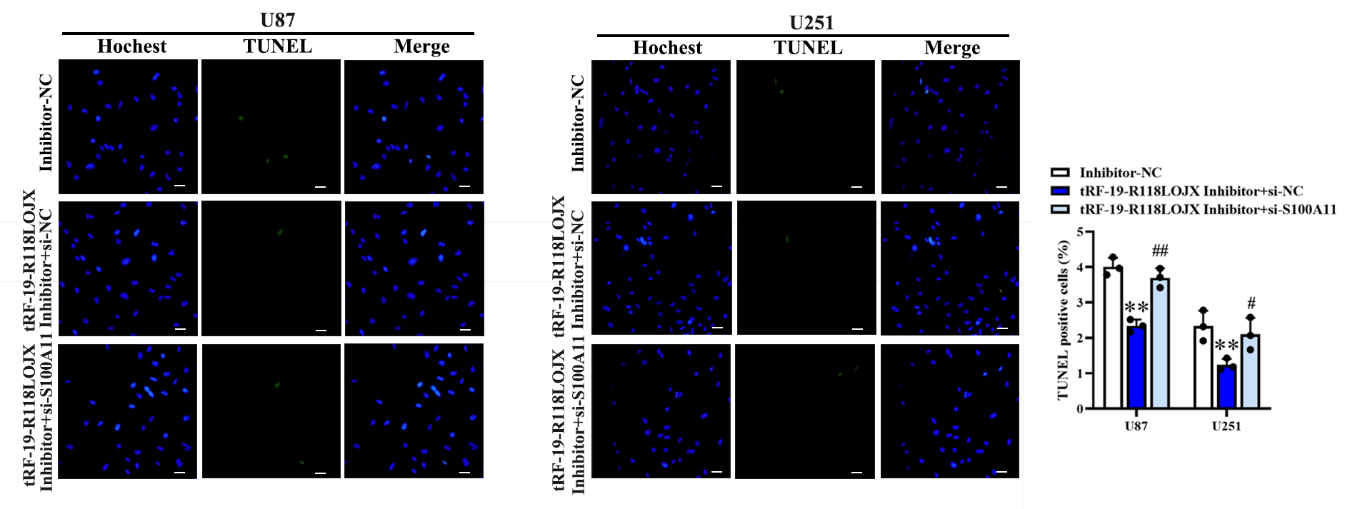


**Fig. S2** The tRF-19-R118LOJX inhibitor inhibits apoptosis of U87 and U251 glioma cells via S100A11. The apoptosis of glioma cells was evaluated by TUNEL assay. (n=3, Scale bars: 20 μm) Data represents mean ± SD, ***P*<0.01 vs. Inhibitor-NC group; ^#^*P*<0.05, ^##^*P*<0.01 vs. tRF-19-R118LOJX inhibitor + si-NC group.


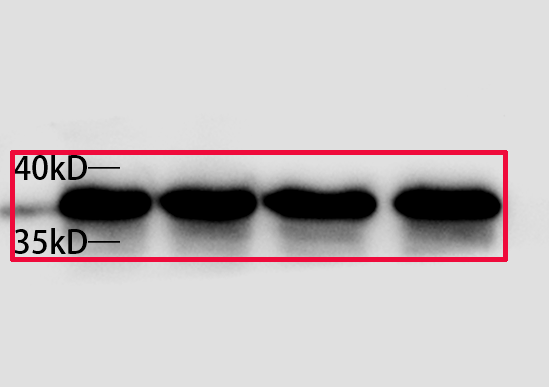


**GAPDH**


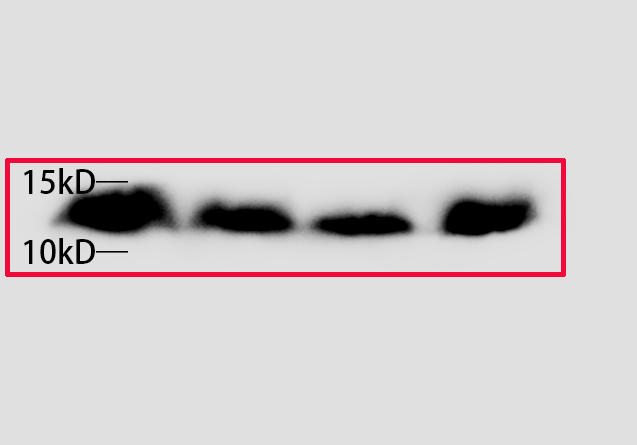


**S100A11**

**Fig. S3** The original images of the western blot in Figure 8A. Since the positions where the protein blots appeared were quite stable and for obtaining clearer bands, we set the upper and lower boundaries of the membranes according to protein molecular weight. All the blots were cropped prior to hybridization with primary antibodies. The red boxes in the original blots indicate the edges of the membrane in Figure 8A of the manuscript.


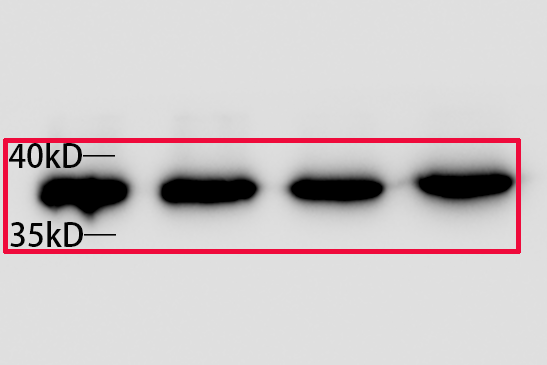


**GAPDH**


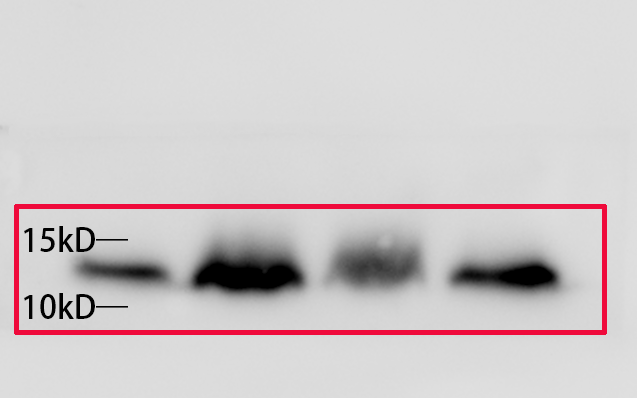


**S100A11**

**Fig. S4** The original images of the western blot in Figure 8B. Since the positions where the protein blots appeared were quite stable and for obtaining clearer bands, we set the upper and lower boundaries of the membranes according to protein molecular weight. All the blots were cropped prior to hybridization with primary antibodies. The red boxes in the original blots indicate the edges of the membrane in Figure 8B of the manuscript.


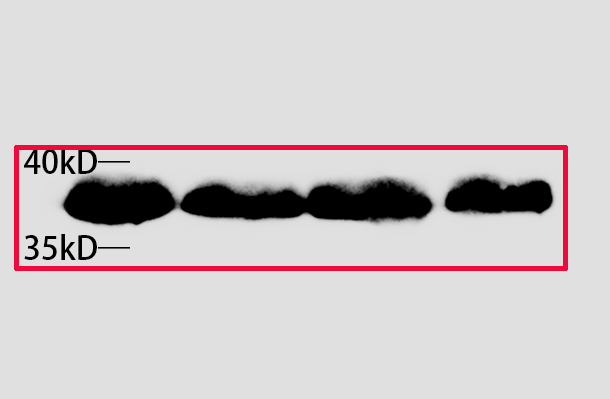


**GAPDH**


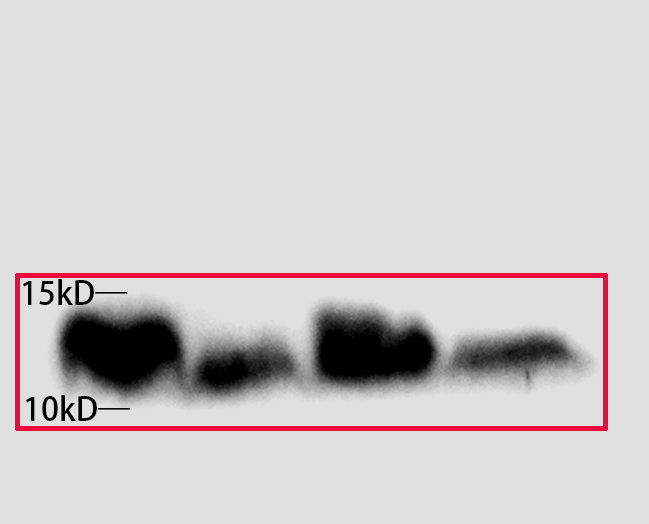


**S100A11**

**Fig. S5** The original images of the western blot in Figure 8D. Since the positions where the protein blots appeared were quite stable and for obtaining clearer bands, we set the upper and lower boundaries of the membranes according to protein molecular weight. All the blots were cropped prior to hybridization with primary antibodies. The red boxes in the original blots indicate the edges of the membrane in Figure 8D of the manuscript.

**U87 cells U251 cells**


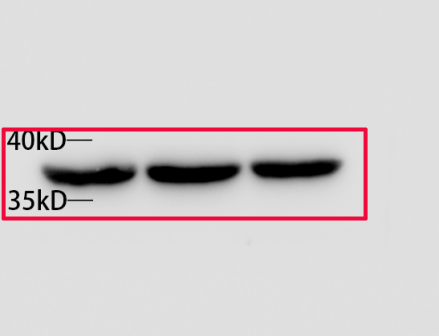

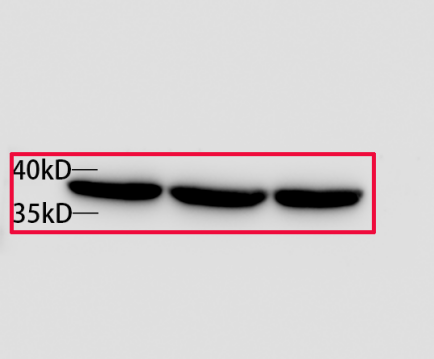


**GAPDH GAPDH**


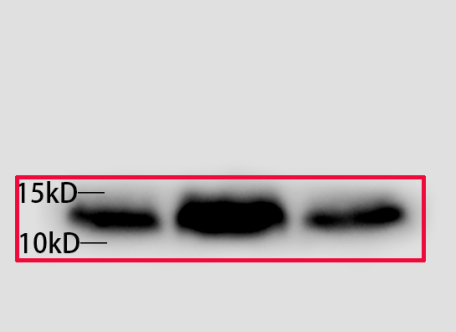


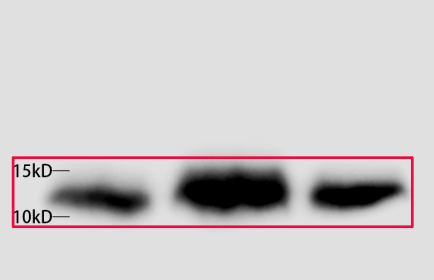


**S100A11 S100A11**

**Fig. S6** The original images of the western blot in Figure 8E. Since the positions where the protein blots appeared were quite stable and for obtaining clearer bands, we set the upper and lower boundaries of the membranes according to protein molecular weight. All the blots were cropped prior to hybridization with primary antibodies. The red boxes in the original blots indicate the edges of the membrane in Figure 8E of the manuscript.
